# Exploring the potential use of Chinese herbs in regulating the inflammatory microenvironment of tumours based on the concept of ‘state-target identification and treatment’: a scooping review

**DOI:** 10.1186/s13020-023-00834-5

**Published:** 2023-09-23

**Authors:** Jing Lian, Dongxin Lin, Yuchan Huang, Xiaohui Chen, Lian Chen, Fan Zhang, Peiling Tang, Jinling Xie, Xiaotao Hou, Zhengcai Du, Jiagang Deng, Erwei Hao, Junhui Liu

**Affiliations:** 1 Guangxi Key Laboratory of Efficacy Study on Chinese Materia Medica, Guangxi University of Chinese Medicine, Nanning, China; 2 Guangxi Collaborative Innovation Center of Study on Functional Ingredients of Agricultural Residues, Guangxi University of Chinese Medicine, Nanning, China; 3 Guangxi Key Laboratory of TCM Formulas Theory and Transformation for Damp Diseases, Guangxi University of Chinese Medicine, Nanning, China; 4 Faculty of pharmacy, Guangxi University of Chinese Medicine, Nanning, China; 5 Department of Bioscience, Faculty of Applied Sciences, Tunku Abdul Rahman University of Management and Technology, Kuala Lumpur, Malaysia

**Keywords:** Tumour inflammatory microenvironment, Inflammatory mediators, State-target identification and treatment, Traditional Chinese herbs, Chinese-western medicine fusion

## Abstract

Tumours do not exist in isolation from the organism; their growth, proliferation, motility, and immunosuppressive response are intricately connected to the tumour’s microenvironment. As tumour cells and the microenvironment coevolve, an inflammatory microenvironment ensues, propelling the phenomenon of inflammation-cancer transformation—an idea proposed by modern medicine. This review aims to encapsulate the array of representative factors within the tumour’s inflammatory microenvironment, such as interleukins (IL-6, IL-10, IL-17, IL-1β), transforming growth factor-beta (TGF-β), interferon-gamma (IFN-γ), tumour necrosis factor-alpha (TNF-α), vascular endothelial growth factor (VEGF), and matrix metalloproteinases (MMPs). Moreover, drawing upon research in traditional Chinese medicine (TCM) and pharmacology, we explore the delicate interplay between these factors and tumour-associated inflammatory cells: tumour-associated macrophages (TAMs), myeloid-derived suppressor cells (MDSCs), tumour-associated neutrophils (TANs) and dendritic cells (DCs). By analyzing the tumour-promoting effects of these entities, we delve into the connotations of Academician Tong Xiao-lin’s novel model of “state-target differentiation” and its application in the diagnosis and treatment of tumours. Our aim is to enhance the precision and targeting of tumour treatment in clinical practice. Delving deeper into our understanding of tumour pathogenesis through the lens of modern medicine, we discern the key etiology and pathogenesis throughout the entire developmental stage of tumours, unveiling the evolutionary patterns of Chinese Medicine (CM) states: heat state → phlegm state → stagnation state → deficiency state. Building upon this foundation, we devised a state-regulating formula. Simultaneously, drawing on pharmacological research in traditional Chinese medicine (TCM), we meticulously identified a range of targeted drugs that effectively modulate the aforementioned tumour-related mediators. This comprehensive strategy—a harmonious integration of state identification, target recognition, and simultaneous regulation—aims to elevate clinical efficacy. The fusion of TCM with Western medicine in tumour treatment introduces novel dimensions to the precise and refined application of TCM in clinical practice.

## Introduction

In the mid-1800s, Rudolf Virchow was the first who correlated inflammation to long-term ailments, like cancer. Inflammatory factors released by the tumours trigger malignant growth, while infections in the body lead to the transformation of healthy cells or obscure tumours into cancer. Since then, numerous researches are on-going to explore the association between inflammation and cancer transformation [1]. Following the rigorous research, the researchers came up with the idea of tumour inflammatory microenvironment, whereby the tumour cells are grown in the environment of inflammatory state. The inflammatory microenvironment provides essential inflammatory substances like cytokines, chemokines, and cytotoxic mediators to promote tumour growth and progression. Hence, scientists are devoted to discovering new strategies to combat tumours by disrupting the tumour’s inflammatory microenvironment. In recent decades, Chinese herbal Medicine and its active ingredients have received extensive attention in the field of precancer prevention and treatment of susceptible populations. Chinese Medicine, as an exceptional medical asset in China, holds distinct advantages in ameliorating the clinical manifestations and biochemical markers of tumour patients [2]. Within the realm of tumour treatment using TCM, the holistic perspective and evidence-based approach play pivotal roles. Nevertheless, it is fundamental to acknowledge the limitations of CM: while the holistic perspective assumes a paramount position in tumour treatment with CM, it occasionally fixates excessively on the prevailing evidence while neglecting a comprehensive grasp of the entire disease process. Moreover, the identification of evidence for disease targeting is relatively feeble, impeding the replication of therapeutic effects and leading to superior individual outcomes but poorer collective efficacy. Consequently, an exploration of novel treatment strategies is indispensable to capture the key disease mechanisms, thereby enhancing clinical precision and therapeutic reproducibility [3].

The concept of “State-target differentiation” was proposed by the esteemed academician Tong Xiaolin, with the intention of drawing inspiration from the physiological and pathological changes in diseases within modern medicine. By incorporating CM perspectives, this concept aims to unravel the disease’s evolutionary trends and comprehensively comprehend its core pathogenesis, consequently establishing corresponding state-regulating prescriptions and medications. Simultaneously, the integration of contemporary pharmacological research facilitates the administration of targeted drugs to amplify disease-specific treatments, thereby achieving a comprehensive therapeutic effect targeting both symptoms and underlying causes. In this context, the term “state” pertains to the dynamic nature, state of affairs, and overall picture of a human body’s internal environment, serving as a comprehensive summary of the core pathogenic mechanisms at various stages of disease development—falling under the macro category. On the other hand, the term “target” refers to the framework of Western medicine, where disease-specific prescriptions and medications are identified based on the aforementioned “state,” thereby targeting the disease itself, its typical symptoms, and relevant clinical physicochemical indicators—falling under the micro category [4].

In our quest to delve into the utilization of the “state-target-cause-effect” therapeutic approach in diagnosing and treating the intricacies of the tumour inflammatory microenvironment, we aim to synergistically integrate the comprehensive insights offered by Chinese and Western medicine regarding the entire trajectory of tumour inflammatory microenvironment development. Furthermore, we shall draw upon the profound wisdom contained within the classifications expounded in the “12th Five-Year” textbook of “Internal Medicine of Traditional Chinese Medicine” [5], cancer is categorized into six categories, namely (i) (Qi stagnation accompanied by phlegm stasis, (ii) heat with toxic congestion, (iii) wet with heat toxicity, (iv) stasis with internal harmful blockage, (v) Yin damage with Qi depletion, and (vi) Qi with Blood deficiency). All of these cancer symptoms are clinically manifested by localized lumps, burning and pain and are closely related to the four characteristics of inflammation, namely redness, swelling, heat and pain, described by Aulus Cornelius Celsus [6]. Drawing upon pertinent literature and clinical acumen, we have aptly grasped the fundamental pathogenic mechanism underlying tumour inflammation. Henceforth, we proffer the epochal conceptualization of the ontological progression of the CM state in the tumour inflammatory microenvironment—comprising heat, phlegm, stagnation, and deficiency. Furthermore, we commence preliminary elucidation on the commensurate state-regulating prescriptions and principal “target drugs” for each stage within the tumour inflammatory microenvironment. Additionally, within the ambits of academician Tong Xiaolin’s paradigm of “state-target differentiation,” we explore strategies for the diagnosis and treatment of tumour inflammation microenvironment [7]. This encompasses multifaceted dimensions: firstly, the use of TCM, which is renowned for its ability to clear heat-toxin, dissipate phlegm and resolve masses, promote blood circulation for removing blood stasis and strengthen vital qi to eliminate pathogenic factor causing the tumour, thus orchestrating the regulation of these peculiar pathological conditions. Secondly, we harness TCM endowed with remarkable anti-inflammatory properties to rectify the manifestations of systemic inflammation. Lastly, with meticulous precision, we employ targeted TCM to intervene in cancerous growths in full accordance with the individualized therapeutic targets. To advance scholarly comprehension, this exposition meticulously scrutinizes representative targeted formulations and distinctive TCM, thereby consolidating a compendium of macroscopic and microscopic approaches in intervening tumours.

## Tumour inflammatory microenvironment

Tumour microenvironment is a dynamic entity that acts like a home for tumour cells development and progression [8]. Tumour cells, host cells, and extracellular matrix (ECM) make up the structure [9]. Besides, strombus tissue, which is made up of fibroblasts, epithelial cells, endothelial cells, inflammatory cells, pericytes, immunological cells, and cytokines are among the other components in the structure [10]. The tumour microenvironment is typically isolated and has a consistent dynamic structure with the features of oxygen deprivation, nutrient deficiency, acidic pH, coagulation, increased interstitial pressure, immunodeficiency, and inflammatory reaction. These environmental features enable the tumour cells to proliferation, differentiate, mutate, infiltrate, migrate, invade, metastasize, adhere, evade immune detection, evade growth inhibitors, an Chinese herbs and form new blood vessels. One of the most significant features of the tumour microenvironment is the inflammatory response. Inflammatory response facilitates the transformation of healthy cells into the cancerous ones, resulting in the increase of TAMs, mast cells, dendritic cells, regulatory T cells, neutrophils, lymphocytes, and MDSCs [11]. The tumour cells infiltrated in the inflammatory mediators, and the inflammatory cells collaborated with the pro-inflammatory cytokines (such as interleukins, TNF-α), growth factors and chemokines to create the inflammatory microenvironment [1].

**Fig. 1:**
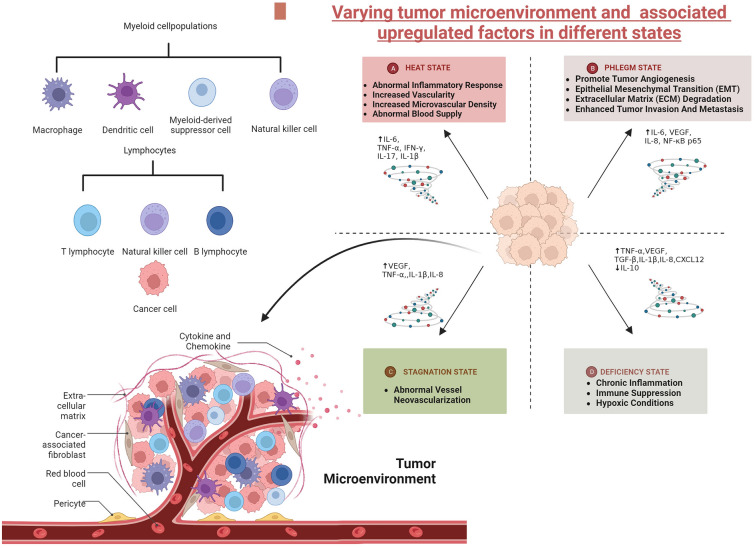
Tumour microenvironment [13–17]

The inflammatory microenvironment serves as a catalyst to transform the normal cells into tumour cells, thus promoting the multiplication and spread. The prolonged release of inflammatory components from these mutated tumour cells keeps the balance of inflammation, which in turns fosters the growth of tumour cells and increases the risk of malignancy. Tumour inflammation exerts a significant impact on the growth and spread of the cancerous cells, hence acts as an essential factor for cancer disease. Figure 1 demonstrates different Tumour microenvironment (TME) and changes of related expression factors in different states of “heat, phlegm, stagnation, and deficiency” [12].

## Inflammatory cancer transformations and the development of tumour inflammatory microenvironment

**Fig. 2:**
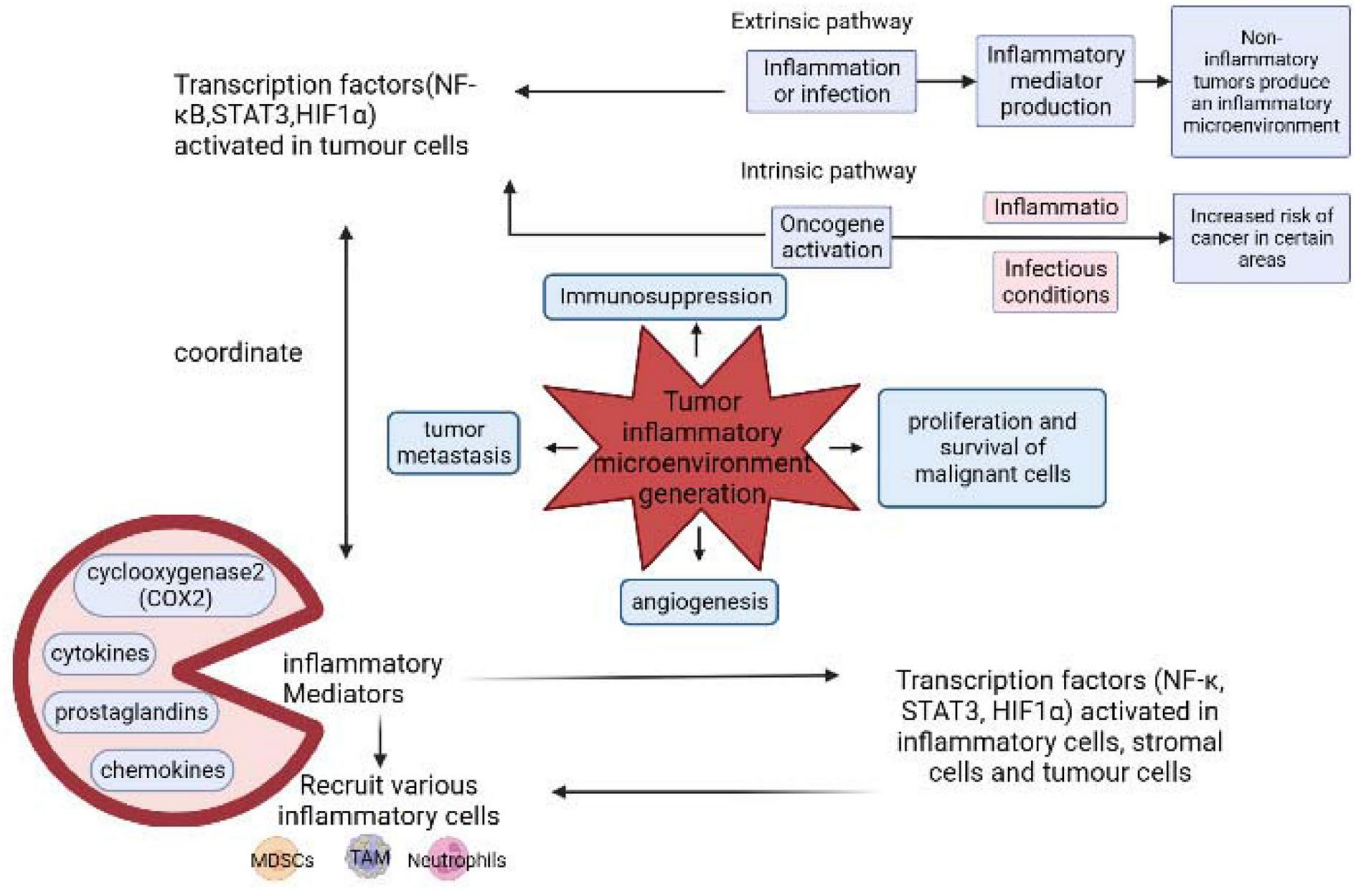
Inflammatory cancer transformation and the development of cancer microenvironment [18]

Cancer can be exacerbated due to the extrinsic factors such as infection, and intrinsic factors like genetic mutations that induce the activation of oncogenes and suppression of tumour suppressor genes, forms a relationship between inflammation and cancer. The dynamic interactions between the extrinsic and intrinsic factors create a convoluted and ever-evolving inflammatory atmosphere, which is regulated by various inflammatory factors released by the infiltrating cells. The inflammatory cancer transformation and the generation development of a cancer-associated microenvironment is shown in Fig. 2. The transformed cells are exposed to tumorigenic genes and produce inflammatory mediators, resulting in the expression of inflammation-related proteins, thus establishing a provocative atmosphere in tumours without the development of an inflammatory state [18, 19].

The inflammatory tumour microenvironment, which is low in oxygen, pH and high in tissue pressure allows tumour cells to thrive. Hypoxia has been proven to suppress the killing capacity of NK cells [20], reduce T cell survival [21] and impede T cell proliferation. Furthermore, hypoxia also impacts the expression of specific markers on the dendritic cells, thus diminishing the efficacy of chemokine receptors and hindering their capacity to activate T cells. A variety of tumour cells thrive under hypoxic and acidic conditions, a study of yeast V-ATPase transfection model by Saroussi and Nelsonrevealed that the activity of the plasma membrane V-ATPase in pumping cytoplasmic H+ from the cytoplasm into the interstitium. This leads to an acidic environment, which in turn leads to tumorigenesis. thus reproduction of NK cells are being suppressed V-ATPase-induced pH changes could trigger the breakdown of the intercellular matrix via Histone Protease B. Thus, promoting tumour occurrence [22]. A high number of tumour cells that had been adapted to the aerobic glycolysis led to the production of large amounts of lactic acid [23]. The acidic environment stimulates the transition of macrophages from M1 to M2, eventually contributes to immune evasion and the development of the disease [24]. Over-accumulation of lactic acid hinders the NK cells from releasing lymphokines, such as perforin and proteases like granzyme, thus reproduction of NK cells is being suppressed [25]. In addition, the tumour induced lactic acid environment reduces the release of CTL cytokines, perforin and granzyme, hence accelerates the spread and metastasis of tumour cells [26]. Moreover, impaired of the performance of anti-cancer immune cells due to high tissue pressure have also been demonstrated in several studies [27].

Tumour cells stimulate a sustained inflammatory reaction and cause the tumour cells to hold under a prolonged chronic inflammatory state, which is a significant component of tumour inflammation. The chronic inflammatory state is essentially unmanageable due to they are influenced by multiple mechanisms and factors within the inflammatory environment. Besides, the irregulated process also causes the accumulation of pro-inflammatory molecules in the inflamed area, hence acts in reciprocation to keep the inflammation continuing, resulting in tumour cells destruction [28].

## The roles of tumour-promoting role of the inflammatory microenvironment in tumours

Several inflammatory factors related to tumours, including interleukins (IL-6, IL-10, IL-17, IL-1β), TGF-β, IFN-γ, TNF-α, VEGF, and MMPs, are also the cancer-associated genes. The release of these inflammatory substances summons and energizes the inflammatory cells such as TAMs, MDSCs, TANs, DCs, NKs, B-cells, and T-cells. The collaboration between these cytokines and inflammatory cells promotes the development and metastasis of cancer cells, genetic mutations, and angiogenesis in the tumour inflammatory microenvironment.

### Inflammatory factors with tumour-promoting effects

#### Interleukin-6 (IL-6)

Studies proved that IL-6, a cytokine with various roles in inflammatory condition, can initiate epithelial–mesenchymal transition (EMT) in tumour cells, and this is evidenced by the decrease of E-cadherin and increase of N-cadherin and vimentin expression which boosting the movement and infiltration of tumour cells [29]. IL-6 triggers the IL-6/STAT3 pathway, and results in the production of CCL2, which further bolsters the potential for the IL-6-mediated EMT events through the expression of twist. The IL-6/STAT3/Twist pathway is critically important in the development of cancer, and impacting tumour cell growth, proliferation, and migration [30].

#### Interleukin-10 (IL-10)

The level of IL-10 expression is closely related to tumour immunosuppression. In the ovarian cancer TME, studies have shown that IL-10 impairs the ability of DCs to stimulate T-cell responses. This is achieved by blocking the expression of IFN-γ, TNF-α, and IL-12, as well as down-regulating MHC class II molecules. Consequently, T cell immunotherapy against tumour growth is inhibited [31].

#### Interleukin-17 (IL-17)

IL-17 is a pro-inflammatory cytokine produced by cells expressing RAR-associated orphan receptor γt (RORγt), including T cells (Th17 cells, a subset of CD4+ T cells) and non-T cells. Research has demonstrated that IL-17 plays a significant role in mediating tumour drug resistance, promoting tumour proliferation, and facilitating metastasis. Its specific mechanisms involve mediating resistance to tumour therapy by enriching bone marrow-derived cells (BMDCs) in the TME, as well as up-regulating the activity of stromal cells [32]. Additionally, in metastatic breast cancer, up-regulation of IL-17 expression promotes MAPK activation, which leads to NF-kB pathway-mediated overexpression of MMP signaling. This, in turn, promotes tumour cell proliferation and metastasis [33].

#### Interleukin-1β (IL-1β)

IL-1β is abundant in the TME and plays a crucial role in promoting tumour growth and inducing tumour immunosuppression. Sequential treatment of wild-type (WT) mice with anti-IL-1β antibodies and anti-PD-1 antibodies has been shown to completely inhibit tumour progression [34]. In non-small-cell lung cancer, the use of IL-1β antibody canakinumab enhances the efficacy of PD-1 and significantly reduces the incidence of lung cancer [35]. IL-1β binding to the IL-1R1 receptor activates the NF-kB pathway, thereby promoting tumour growth. Furthermore, in triple-negative breast cancer, IL-1β stimulates TAMs recruitment, driving tumour growth and immunosuppression [36].

#### Transforming growth factor-β (TGF-β)

Evidence shows that TGF-β pathway is a suppressor of tumour growth in the context of the tumour environment [37]. Despite this, if cancer causes changes in the components of the TGF-β pathway, the action of the particular components are disrupted, eventually the cancer cells are being refrained from the suppression by TGF-β. Several studies revealed the correlations between mutations of SMAD family proteins (Smad2, Smad3, Smad4 and Smad7) and various diseases and cancers [38], with the intracellular signal transduction molecule proteins (SMAD) in the TGF-β pathway. Various forms of gastrointestinal malignancies, including colorectal, pancreatic, lung, and squamous head/neck carcinomas, are among the cancers that associated with the decrease of the expression of anti-cancer genes Smad2 and Smad4 which function to control the transcription and suppression of tumour growth. On the contrary, lack of term due to mutations resulted the reduction of cancer-fighting capacity of TGF-β pathway. Smad3 is often rendered inactive in the malignant haematological conditions, which leads to a more significant proliferation of cancer cells [39]. In addition, the inhibitory Smad (I-Smad) in the Smad family that impedes signaling, and Smad7, which is a specific I-Smad, that acts as a signal inhibitors in the TGF-β signaling system [40] act to impede transmission. In contrast, pancreatic cancer may cause an increase in Smad7 expression, which leads to a gradual decline in cell health. TβR-II, a gene known to possess the cancer-combating properties participating as part of the TGF-β signaling cascade [39] and its decreased expression are associated with in contrarily, pancreatic cancer lack of term due to mutations h the malignant transformation of tumours. Furthermore, shift mutation in the 10th base pair of Exon 3 of TβR-II is commonly reported to alter the receptor, finally affects the TGF-β cascade, and resulting in a decrease of tumour control, and increase the chance for the tumours to refrain from the TGF-β cascade, thus increases the metastatic capacity of the cancer cells [39].

#### Interferon-γ (IFN-γ)

In 1965, Wheelock made a pioneer discovery on the antiviral substance IFN-γ, which is the part of the type II interferon family and is a cytokine that stimulates an inflammatory response initiated by T cells and NK cells. Despite the initial evidence suggesting that IFN-γ could promote tumour immunity and antiviral activity, results of clinical trials reported in contrary. IFN-γ was reported to induce tumour immunosuppression and pro-tumour growth when used in combination with carboplatin/paclitaxel, thus resulting in shorter survival times for ovarian cancer patients [41]. IFN-γ appears to exhibit antagonistic effects on chronic inflammation and the prolonged infiltration in the tumour microenvironment [42]. The presence of the molecules belonging to the primary Histocompatibility Complex Class I (MHC I) and CTL-associated proteins may be altered by the tumour cells, leading to the inactivation or loss of MHC I and CTL [43]. This mechanism would reduce the responsiveness of tumour cells to the IFN-γ-mediated immune effects, eventually accelerating the immune escape. Conversely, simultaneous activation of Src homologous tyrosine phosphatase (SHP2) and suppression of cytokine signaling (SOCSs) by the tumour cells [44], in addition to the IFN-γ downstream molecular activator of transcription (STAT) impede the IFN-γ immune surveillance process, subsequently preventing the tumour cells from being detected. Furthermore, the IFN-γ can also either foster the tumour cell expansion directly or indirectly. The level of IFN-γ in the tumour cells is critical, as too much will accelerate the tumour growth and spread, whereas too low will lose its desired effects. Besides, IFN-γ has also been observed to strengthen the endurance of malignant cells by altering the integrity of their genome [45], thus making them more resilient to cancer treatment.

#### Tumour necrosis factor-α (TNF-α)

It has been proven that TNF-α fosters tumour proliferation [46]. The primary factor in the breast cancer microenvironment is TNF-α, which is an important pro-inflammatory cytokine, primarily released by the tumour-associated macrophages and the cancerous cells. The progression of breast cancer is linked to TNF-α, which causes the mesenchyme transition, and the spread and proliferation of the disease [47]. Additionally, previous research has also suggested a positive correlation between TNF-α and colorectal cancer due to its ability to increase COX-2 expression, damage DNA, and stimulate tumour angiogenesis [48]. Furthermore, TNF-α has also been found to regulate SOD-2 and promote tumourigenesis in an experimental rodent system with inflammation-driven lung adenocarcinoma [49].

#### Vascular endothelial growth factor (VEGF)

Non-small cell lung cancer (NSCLC) is a prevalent and serious type of lung cancer. In NSCLC, the role of VEGF and its receptor is significant in promoting an immunosuppressive tumour microenvironment. This occurs through direct effects on both innate and adaptive immune cells, as well as indirect effects on endothelial cells [50]. Consequently, this leads to the development of drug resistance and a poor prognosis for the tumour [51]. One specific mechanism by which VEGF contributes to the immunosuppression of NSCLC is by mediating tumour microangiogenesis and activating antigen-presenting cells, regulatory T-cells (Treg), and tumour-associated macrophages. These processes collectively contribute to the immunosuppressive environment within the NSCLC tumour [52].

#### Matrix metalloproteinases (MMPs)

Matrix metalloproteinases (MMPs) are a class of proteases closely associated with tumour cell infiltration and metastasis. They play a key role in degrading and altering the extracellular matrix structure, allowing cancer cells to invade surrounding tissues. Research has shown that the key substances regulating the process of matrix degradation are the endogenous inhibitors of MMPs, known as tissue inhibitors of metalloproteinases (TIMPs). In normal cells, MMPs are involved in the degradation and remodeling of the extracellular matrix, maintaining a balance. However, in cancer, the activity and regulation mechanisms of MMPs are often imbalanced, thereby promoting tumour infiltration and metastasis [53]. It is worth noting that the importance of MMPs in the tumour inflammatory microenvironment should not be overlooked. The tumour inflammatory environment can lead to overexpression of MMPs, which disrupts the extracellular matrix structure. In addition, in breast cancer patients, chronic inflammation can upregulate the expression of MMPs (such as MMP-2 and MMP-9), thus promoting tumour angiogenesis, tumour occurrence, and metastasis [54]. Overall, MMPs and their regulation play a significant role in tumour biology. Understanding the role of MMPs and their interactions with other factors is crucial for developing effective strategies to inhibit tumour invasion and metastasis.

### Inflammatory infiltrating cells that promoting tumour growth

#### Macrophages (TAMs)

TAMs can be classified into M1-type, which exhibits the anti-tumour effect, and M2-type, which exhibits the pro-tumour effect [55]. The transition of M1-type into M2-type in the progression of tumour cells caused the increase of M2 macrophages and associated with augmenting tumour angiogenesis, enhancing tumour invasion and metastasis, inducing drug resistance and suppressing tumour immunology [55–57]. M2 macrophages secrete various cytokines such as MMP-9, MMP-2, MMP-12,VEGFIL-8, IL-1 and fibroblast growth factor to induce tumour angiogenesis and a worsened prognosis [58]. In addition, M2 macrophages also in the meanwhile, the tumour cells, activate the STAT3 transcription activator to stimulates tumour proliferation by secreting inflammatory factors such as IL-6,TNF-α, TGF-β, and fibroblast growth factor, as well as the degradation of extracellular matrix components, which facilitate tumour cell invasion and metastasis. Furthermore in breast cancer cells, M2 macrophages enhance tumour resistance to antitumour drugs like paclitaxel by inhibiting Caspase3 apoptotic signaling [59]. Finally, M2 macrophages also inhibit the tumour immune response mediated by killer T cells and natural killer cells [60].

#### Myeloid-derived suppressor cells (MDSC)

MDSCs derived from immature and undifferentiated cells which can be categorized into G-MDSC and M-MDSC [58]. MDSC infiltrated by inflammatory microenvironment are closely related to tumour immunosuppression and tumour cell invasion and angiogenesis, and immunosuppression of MDSC is closely related to a variety of immune cells: when the supply of l-arginine is deficient, MDSC will inhibit t cell production and thus inhibit the immune response [61–63]. M-MDSC can achieve tumour immunosuppression by inducing NO production through the high expression of INOS [64]. MDSC can also inhibit IFN-γ secreted by NK cells and inhibit its cytotoxicity to achieve immunosuppression [65]. MDSC in the tumour environment can achieve immunosuppression through the high expression of protein enzymes such as MMP high expression to promote tumour invasion and progression [66]. MDSC can also participate in tumour angiogenesis by mediating the migration of endothelial progenitor cells in the circulatory system through the secretion of MMP-9.

#### Tumour-associated neutrophils (TANs)

Neutrophils have both anti-tumour N1 type and pro-tumour N2 type behaviours. Fridlender et al. found their pro-tumourigenic effects were associated with inhibition of the N1 phenotype and activation of the N2 phenotype by TGF-β [67]. and TGF-β could induce polarization from N1 to N2 [68]. and was associated with type I interferons [69] and their pro-tumourigenic effects were also associated with reactive oxygen species (ROS), cytokines, chemokines, and extracellular reticulum traps. Neutrophils are able to release ROS to inhibit the immune response of T cells, and also upregulate the secretion of TGF-β, CCL4, and CXCL8 to promote tumour growth. NETs were found to promote tumour proliferation by activating the NF-κB pathway, and to utilize the tumour cell trapping function to support early tumour cell adhesion and promote tumour metastasis [70].

#### Dendritic cells (DCs)

Dendritic cells (DCs) play a key role in anti-tumour immune responses [71]. It was found that in the TME, DCs are usually in an inactive state, which may limit the adaptive immune response induced by tumour growth [72]. The TME affects the immune function of DCs, in which hypoxia and ROS generation may be one of the important factors. On the one hand, hypoxia and ROS can inhibit the maturation process of immature DCs; on the other hand, they can also lead to a more migratory and inflammatory DC phenotype [73]. It has also been shown that myeloid-derived suppressor plasma cells (MDSC) have an inhibitory effect on tumour immune functions such as immune surveillance and antigen presentation of DCs in tumour cells, and also affect the maturation process of DCs of the myeloid lineage [74].

## State-targeted identification of herbs on the intervention of inflammatory microenvironment of tumours

### Treatment strategy of “state-target identification and treatment”

Academician Xiaolin Tong developed the “Target Identification and Treatment” strategy through the combination of CM and modern medical techniques. CM holds the holistic notion in high regard, and viewing a healthy body must be in a constant equilibrium. If this equilibrium is being disrupted, the body’s internal environment will be imbalanced, and potentially develop various diseases. Malignant tumour is a long-term disease that disrupts the body’s equilibrium, and swelling plays a pivotal role in their expansion. Tumour inflammation is responsible for the disruption of the microenvironment.

CM treatment focuses on rectifying any imbalances in the body and restoring its internal equilibrium to combat diseases.

The “state-target differentiation” strategy is a medical innovation system that combines the theories of CM and modern medicine proposed by Academician Tong Xiaolin. CM emphasizes the concept of wholeness and believes that a healthy human body is in a state of dynamic balance. When the balance is broken, the internal environment of the body is disturbed, and it is easy to be attacked by diseases, and various “distortions” appear. Malignant tumour is a chronic disease with imbalance of homeostasis, and inflammation is one of the important factors for tumour development. Tumour inflammation microenvironment is a manifestation of microenvironmental imbalance caused by tumour inflammation.

According to the theory of “state-target differentiation,“ the treatment of malignant tumours can be divided into four stages. In the early stage, evil qi invades. In the middle stage, there is an interplay between good and evil. In the late stage, cancerous toxins gradually penetrate into the body, resulting in a deficiency of positive qi. Scholars who start from the perspective of inflammation have discovered a close relationship between tumours and abnormal secretion of inflammatory factors as well as immune imbalance. They propose the theory of inflammation-cancer transformation, which suggests that the formation of cancerous toxins is closely related to heat, phlegm, stasis, and deficiency. The process includes the transformation of heat into phlegm, phlegm into stagnation, and finally into positive deficiency. Under the influence of dampness and heat, they interact and intensify each other. Dampness becomes hotter due to heat, leading to the formation of Damp-Heat Proof. Over time, damp-heat evil qi further disrupts the body’s fluid balance, causing damp-heat to transform into phlegm. The hot dampness burns the fluids and forms phlegm, obstructing the flow of qi and blood. Eventually, the combination of phlegm, dampness, and heat gradually results in stagnation. Blood stagnation hinders the normal circulation of qi and exacerbates the disease’s progression. Ultimately, the prolonged effects of damp-heat evil and stagnation weaken the positive qi, damaging it and causing a progressive decline. This weakened positive qi state accelerates tumour development and worsens the disease. At this stage, toxins have deeply penetrated the bloodstream, significantly escalating the severity of the disease. Phlegm evil disrupts normal blood flow and combines with dampness and heat evils, gradually manifesting as palpable lumps.

**Fig. 3:**
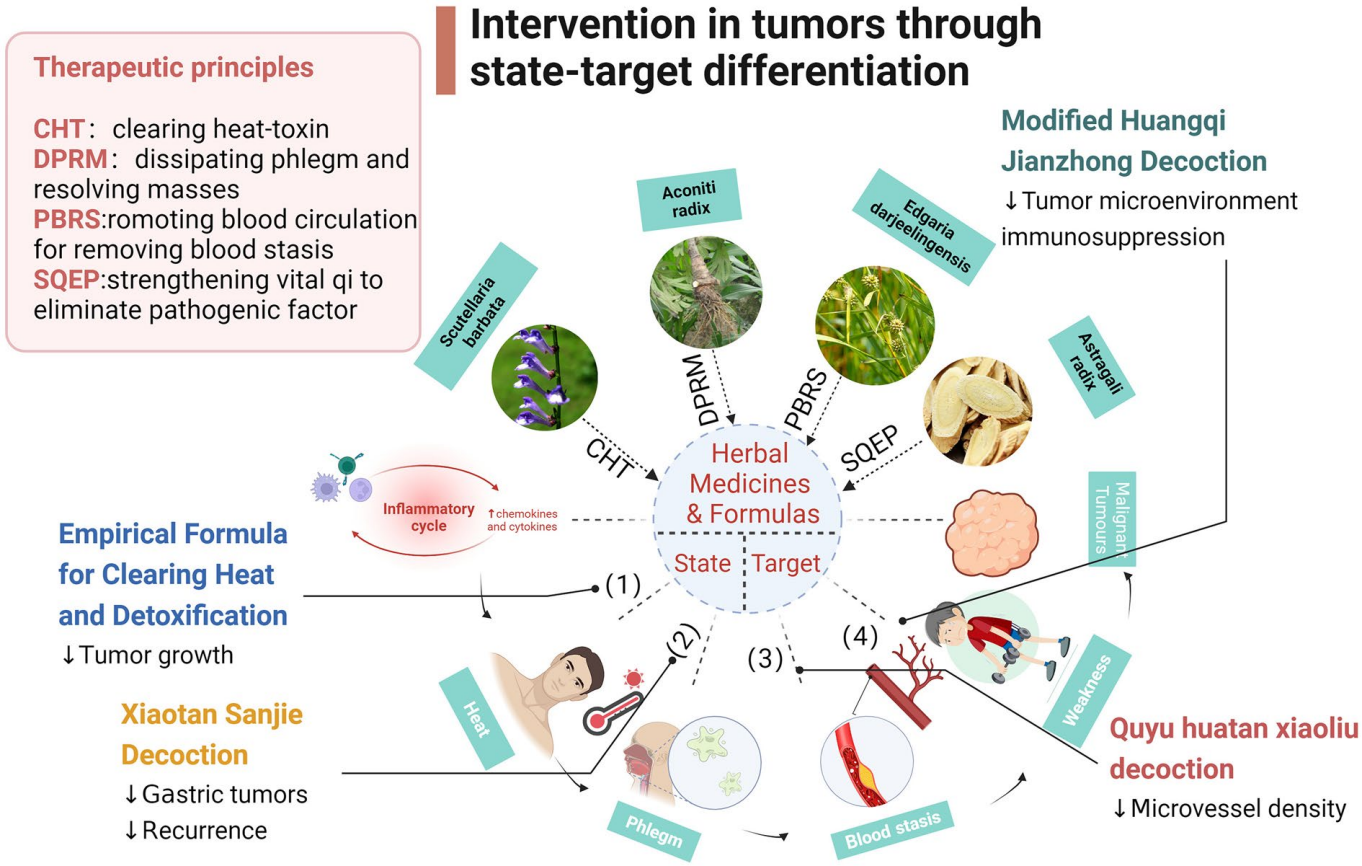
Intervention in tumors through state-target differentiation [78–81]

To maintain the balance of yin and yang in the body, various treatments can be utilized to address the aforementioned imbalances. These may include heat-clearing and detoxifying therapies, phlegm elimination and stagnation dispersal techniques, blood circulation promotion and blood stagnation elimination methods, as well as positive support and dispersal of evil spirits. Representative formulas, such as prescription of Qingrejiedu [75], Xiaotan Sanjie Decoction [76], Quyu huatan xiaoliu decoction [77], and Modified Huangqi Jianzhong Decoction [78], can be selected to regulate these different conditions. Moreover, in order to target inflammation, anti-inflammatory herbs like *Hedyotis diffusa* and *Asarum sagittarioides* can be employed to alleviate the body’s inflammatory response. To specifically address tumor-related physiological and chemical indicators such as IL-6, TGF-β, IFN-γ, TNF-α, MMPs, and others, Chinese herbs and representative formulas that demonstrate specific effects on these indicators can be sought as interventions for tumors at both macro and micro levels (Fig. 3).

### Tuning “state”

CM is unique in providing a comprehensive assessment on the progression of the disease based on its staging system. Compared to the Western approaches, CM emphasizes on the maintenance of the equilibrium between human body and environment, relying on the holistic notion of harmony between Heaven and Earth, and yin and yang. The Treatments are thus approached to a more macroscopic viewpoint [82]. By taking into consideration the external environment and attempting to understand the internal conditions, the manifestations of the disease can be under controlled, and the inclination of the disease can be adjusted to enable the body to maximize its capacity to self-heal [83]. Through CM approach, equilibrium between yin and yang of the body is the ultimate goal for the identification and treatment of the disease states. After diagnosis, the process of pinpointing symptoms can be omitted, and the target can be directly added into the prescription to adjust the disease’s state.

Among the CM professionals, cancer development is widely perceived as a result of an imbalance between good and evil forces [84]. The “Treatise on the Origin of Diseases—Cumulative Diseases” stated that all organs in the body are regularly impacted by the negative. In the beginning, they are unable to form a barrier, yet when they become stagnant, they accumulate lacking beneficial energy that can contribute to the growth of tumours. This deficiency causes the body produce metabolic toxins such as stagnant blood, phlegm, and cancer toxins. Over time, these elements combine and form tumours. Excessive heat, inactivity, stagnation, and deficiency cheat damages the body, causing an increase in a conducive environment for the development of heat in the body, with the Yang heat often being the primary source of inflammation. The heat from the fire can damage the vessels, impair the flow of fluids, and produce phlegm, and lead to pathological changes such as congestion, deficiency, and ultimately tumour growth. The four states of heat, inactivity, stagnation, and poverty should be regulated to treat the microenvironment of tumour inflammation, according to the principles of CM state-target identification and treatment. Chinese herbs can be used to cool down the body, eliminate toxins, reduce mucus, break down obstructions, improve blood flow, and reduce blockages to sustain equilibrium in the cells and alleviate tumour inflammation. The herbs act to enhance the four conditions of warmth, mucus, stasis, and inadequacy for impeding tumour proliferation and promoting the patient’s well-being.

#### Heat state

CM believes that abundance of negative energy in the body over an extended period can cause tumours. Heat damages the body, causing an increase in fluids and mucus, a decrease in Qi and blood flow, and blockages of organs and channels due to the heat and toxins released by the blood clot, mucus, and impurities. The blood clots, mucus and impurities can cause sores and lesions in the body. “The Golden Mirror of Medicine” states that carbuncles and gangrene are caused by the toxic heat that blocked the meridians, which results in the stagnation of both blood and Qi. The literatures of both antiquity and modernity indicate that heat toxicity causes tumours [85]. Patients who are suffering from the internal heat toxicity due to the body’s heat state can take the heat-clearing herbs, such as Lonicerae Japonicae Flos, Hedyotis Diffusa, Lobelia Chinensis Herba, Scutellaria Barbatae Herba, Forsythiae Fructus, Sophora Tonkinensis Radix et Rhizoma, Isatidis Radix, Coptidis Rhizoma and Phellodendri Chinensis Cortex, to regulate the heat state at the macro level and reduce the symptoms such as burning, thirst, body heat, constipation and pain. Recent pharmaceutical research demonstrated that the above mentioned herbs can efficiently impede tumour growth, prompt tumour cell death, and modulate the immune system [86].

#### Phlegm state

The text “Jing Yue Quan Shu” warns that if one’s diet remains stagnant, it may impede progress and will not yield the desired outcome [87]. In the initial stage of tumour growth, the body is affected by six malfunctions that involve phlegm, as well as dampness infiltration. The weakened spleen yang can impede the flow of qi, promote mucus buildup, and cause lumpy formations. Moreover, an unhealthy diet, either too heavily seasoned or invigorating, can negatively affect the spleen and stomach [88] capacity in transferring and processing nutrients, consequently limit the amount of moisture and result in the accumulation of phlegm. Chinese herbs like Coicis Semen, Polypous, Poria, Melismatic Rhizoma, Bombyx Batryticatus, Pinellias Rhizoma and Arisaematis Rhizoma can be employed to boost immunity, limit tumour development, and adjust the patient’s body’s moisture and phlegm buildup.

#### Stagnation state

Patients with tumour typically display a hypercoagulable shape due to the increased blood viscosity. However, CM views it as a symptom of the body’s fluids depletion due to the excessive evil heat generated by inflammation, thereby causing the blood to become stagnant and slow down the circulation. This concept is now widely accepted by modern medicine, whereby the generation of an inflammatory reaction activates the emission of cancer-related substances and triggers the rise of inflammatory mediators, which trigger the external clotting system to amplify the thrombin level [89]. Therefore, the balance of blood flow and clotting is a critical element in tumour genesis and progression, as well as a significant contributor to the poor health of cancer patients. The CM prescribed Herbal medicines that activate the blood flow and break up the stagnant blood could be a powerful way to modify the environment of the tumour-related inflammation and prevent further growth of the cancerous cells.

#### Deficiency state

The “Jing Yue Quan Shu” states that the key of treating the tumour’s progression is to recognize the appropriate timing of intervention and supplement energy. If the accumulation is persisted for a prolonged period, the vital energy will become increasingly feeble, rendering it unwise to launch an attack, as the stomach is near to the gas and would be the first to suffer from the harm. The more the patient strikes, the weaker the patient [90]. The lack of beneficial energy is the significant contributor to tumour growth. If the body lacks positive energy, Yin and Yang can turn imbalance the qi and blood flow disrupted, metabolic issues are being triggered, and the internal environment is being altered. Herbs that bolster positive energy and reinforce the whole being should be consumed to replenish the positive energy that is lost during the battle against the evil force, so that the body can be fortified against the wickedness and the patient can be alleviated from the deficiency due to the tumour. Research conducted in the field of clinical pharmacology has proven that herbs such as Ginseng Radix et Rhizoma, Astragali Radix, and Ophiopogonis Radix can increase the circulation of qi and blood in the body, balance the yin and yang, and unblock the internal organs. In addition, these herbs may also effectively regulate the immune system, suppressing tumour growth, invasion, and metastasis, inducing apoptosis, and inhibiting the formation of tumour blood vessels.

### Target shooting

When dealing with the cancerous growths, addressing the underlying cause is paramount, and in the case where the tumours are caused by inflammation, the source of the inflammation must be identified. As inflammation is the primary target of the disease, CM believes anti-inflammatory treatments and immediate improving the microenvironment of tumour inflammation will be an effective strategy. By using herbs with anti-inflammatory effects, inflammation conditions in the cancer patients can be reduced, the inflammatory environment can be regulated, the pain can be reduced, and eventually immunity can be enhanced. The goal of controlling blood pressure in the patient is to address the four primary pathogenic conditions of heat, phlegm, stasis, and deficiency for malignant tumour progression by employing a combination of medications and formulas. Western medicine focuses on treating the symptoms of blood pressure by specifically targeting the blood pressure. To optimize the treatment of malignant tumours using Chinese herbs, it is essential to determine the most efficient medications for this purpose. This paper takes into the account of the recent pharmacological findings in Chinese medicine, whereby herbs that are targeting to the release of cytokines like IL-6, TGF-β, IFN-γ, and TNF-α are focused, then employs in combination with the evaluation methods of Western medicine and CM to achieve precise disease management.

Various inflammatory molecules like IL-6, TGF-β, IFN-γ, and TNF-α are associated with tumour development, while tumour recurrence is associated with its spread [91]. Additionally, the vascular endothelial stimulating hormone, which accumulates at the site of injury [91–93], was found to implicate in the process of tumour immune evasion, tumour expansion, and metastasis. Currently, oncology research is focused on exploring the ways to alter the tumour microenvironment and identify targeted treatments.

### Moderate hitting target herbs

Herb used in Traditional Chinese Medicine, such as scutellarin barbate herba, are proven to effectively control the body’s heat state, which is a significant factor that causes tumours. Controlling the body’s heat state can reduce abdominal mass, bloating, and pain in the patients with colorectal cancer, and lower AFP levels in the liver cancer patients. The active ingredient, galbanum polysaccharide, is proven to effectively hinder the immunosuppression caused by the tumour inflammatory microenvironment. The study conducted by Ye Hua revealed that galbanum polysaccharide disrupts the immunosuppression of the tumour by altering the ratio of cytokines in the TH1/TH2 subgroup of the tumour microenvironment when it was administered to the serum of C26 tumour-bearing mice [94]. Furthermore, found out that sempervivum’s aqueous extract not only restricted tumour angiogenesis in the S180 mice’s tumour microenvironment, but also promoted DC infiltration in the mice to reinforce the immunity [95]. The study proved that sempervivum’s aqueous extract exerted both angiogenesis-inhibiting and immunity-stimulating effects on the tumour inflammatory microenvironment. Herb like Artesunate, which is derived from artemisinin (ARS) and dihydroartemisinin (DHA), has been proved to reduce the transmission of TGF-β signaling pathway, and reversed the tumour expansion and diffusion which have been set off by the CAFs in breast cancer [96].

In addition, herbs like Coicis Semen can be utilized to reduce VEGF expression, hence preventing the growth of tumours, and treating dampness accumulation and phlegm congestion in cancer patients. Cervical cancer patients have reported an improvement in stool hardening. The Weakened spleen yang, laxity, and loose stools after prescription of this herb. In addition, this herb also reduced abdominal distension in liver cancer patients and caused a marked decrease in the abnormal CEA index in gastric cancer patients. Furthermore, Xu et al. discovered that Coix lachrymal oil efficiently reduced the generation of VEGF and bFGF, a basic fibroblast growth factor, in the human pancreatic cancer on site BxPC-3 cells, which affected the cell growth cycle and prevent the growth of new blood vessels for the survival of tumours [97]. In spite of that, Liu Fang et al. found out that sashimi extract potentially decrease the formation of blood vessels in the H22 mice with hepatocellular carcinoma [98].

Studies have shown that herbs that stimulate blood flow and disperse stagnation can reduce tumour growth. When taken together with the herbs that invigorate qi, the efficacy of the herbs in preventing tumour spread and augmenting immune response are improved [99]. Zhou et al. discovered that administering the targeted anti-cancer formula to activate blood circulation had a positive influence on the NK cell killing ability [100], besides stimulating IFN-γ emission from the T cells in the H22 liver cancer mice. With this, the body’s immune response can be significantly improved and the tumour size reduced. Besides, *Salvia miltiorrhiza* which contains Tanshinone, has been found to effectively impede the progression of hepatocellular carcinoma in mice by suppressing the release of TGF-β [101], increasing the expression of p38, causing apoptosis, and ultimately limiting the tumour growth, thereby prolonging the survival of the mice.

Herbs which are known to nurture and sustain are widely used in CM to bring up the beneficial energy and maintain the equilibrium of yin and yang, to improve the body’s immunity, and mitigate the insufficiency of the cancer patients. Taking Ginseng Radix et Rhizoma as an example, Wang Lei et al. reported that ginsenoside Rb1 could counteract the TGF-β1 cytokines released by the liver cancer cells, thereby preventing the NK cells from being immunosuppressed [102]. Du et al. unveiled ginsenoside Rg3 could disrupt the proportions of MMP-9 and matrix metalloproteinase-inhibitor-1 (TIMP-1) in the HT-29 cell line’s extracellular matrix, obstructing tumour cell infiltration and metastasis, resist TGF-β then obstruct the increase of VEGF and MMPs expression, hinder tumour angiogenesis and obstruct the tumour inflammatory microenvironment [103]. Besides, Ginsenoside Rg3 also reduces the expression of PD-L1, thereby allowing the T cells to combat immunosuppression better and reducing the expression of tumour angiogenesis-associated genes such as EGF and MMP-1 [104]. In spite of that, Ginsenoside Rh2 was also proven to transform the macrophage phenotype from M1 to M2, thereby decreasing VEGF, MMP-2, and MMP-9 expression, eventually inhibit the spread and enlargement of tumours [105]. These findings reflect the principle of Chinese medicine of ‘treating the untreated’ and highlight the importance of herbal medicine in tumour treatment research.

**Table 1:** Effects of Chinese medicine on tumor inflammation and cancer models

TCM		Antitumor inflammatory microenvironment effects	Types of cancer	Cancer models	References
Chinese herbal components	Icaritin	↓IL-6/JAK2/STAT3 signaling pathway	Multiple myeloma	MM xenograft mouse models	[106]
	Ginsenoside Rb1	↓TNF-α and IL-6	Cancer cachexia	C26 cancer cachexia mouse	[107]
	Notoginsenoside R1	↓IL-6↓JAK2/STAT3 signaling pathway	Osteosarcoma	U2OS osteosarcoma cells	[108]
	Astragaloside	↓IL-6/STAT3 signaling pathway ↓IL-6R, STAT3, survivin, and cyclin D1	Colitis-associated cancer in mice	AOM/DSS mouse model	[109]
	Platycodin D (PD)	↑IL-2, IL-6, TNF-α and IFN-γ	H22 hepatocellular carcinoma	Murine solid tumours H22 transplanted model	[110]
	Esculentoside A (EsA)	↓ALDH1A1, Sox2, and Oct4↑proapoptotic proteins, Bax and cleaved caspase-3↓Bcl‐2↓IL‐6/STAT3 pathway	Breast cancer	Murine breast CSCs (EMT6M)	[111]
	Icariin (ICA)	↓TGF-β1, TNF-α, IL-6, IL-17A, IL-10↓Ki67, survivin, Bcl-2, c-Myc↑P16, P53, Bax	Cervical cancer	Mice bearing U14 cervical tumour	[112]
	Aloe emodin	↓JNK↓IL-1β and IL-6↓VEGF and MMP	Breast cancer	4T1 breast cancer cells	[113]
	Osthole	↓MMP-9 and vimentin	Liver cancer	Human liver cancer HepG2 cells	[114]
	Osthole	↓M0-M2↓MRC1, CCL22 and TGF-β↓p-STAT6 and the p-ERK1/2-C/EBP b axis	Pancreatic cancer	C57BL/6 mice model	[114]
	Matrine	↑IFN-γ/TNF-αand IL-p70	Human gastric carcinoma	The cytotoxic T lymphocyte (CTL)	[115]
	β-Glycyrrhetinic acid	↓I-κBα和NF-κB↓TNF-α	–	RAW 264.7 cell	[116]
	Baicalin	↓MMP- 2 and MMP- 9↑TIMP- 2↓TLR4, p- IκBα, and NF- κB p65↓TLR4/NF- κB signaling pathway	Colorectal cancer (CRC)	Subcutaneous xenograft tumour mouse model of CT26 cells	[117]
	Curcumin	↓MDSCs↓TLR4/NF κB signaling pathway↓IL 6, IL 1β, prostaglandin E2 and COXe 2	Liver cancer	HepG2 xenograft model	[118]
	Curcumin	↓NF-κB↑TRAIL receptors↑peripheral mononuclear neutrophils	Bladder cancer	MBT-2 murine tumour models	[119]
	Tanshinone IIA, xanthohumol, and curcumin	↓PI3K/AKT signaling pathway, BCL6, VEGF	Glioma	–	[120]
	Matrine	↓PI3K/AKT/mTOR signaling pathway	Lung cancer	A549 and 95D lung cancer cells	[121]
Chinese herbal formula	Empirical formula for clearing heat and detoxification	↓TAMs	Endometrial cancer	Xenograft tumor of endometrial carcinoma	[79]
	Taohong Siwu decoction	↓EMT	Breast cancer	HER-2+ breast cancer	[122]
	Eight-treasure decoction	↓hs-CRP, ↓IL-6, IL-8	Breast cancer	–	[123]
	Huatan Tongyu Jiedu recipe	↓Micro-vessel density and ↓VEGF	–	Mice bearing S180 transplanted tumor	[124]
	Huatan Xiaoliu decoction	↓VEGF, ↑nm23 mRNA	Lung cancer	Lewis tumor-bearing mice	[77]
	Xiaotan Sanjie decoction	↑RUNX3↓NF-κB p65	Gastric cancer	Human gastric cancer cell SGC-7901-bearing nude mice	[125]
	Huayu Jiedu Tongluo recipe	↓COX-2↑PGI, G-17; ↓IL-1β, IL-8, TNF-α	Stomach cancer	Stomach-collateral stasis-toxin type gastric precancerous lesion	[126]
	Yiqi Huayu Jiedu prescription	↓HIF-1α, VM	Hepatocellular carcinoma	Human hepatocellular carcinoma sorafenib resistant cells in nude mice	[127]
	Modified Huangqi Jianzhong decoction	↓CXCL12/CXCR4 signaling pathway	Lung cancer	Mice bearing Lewis lung cancer with syndrome of spleen qi deficiency	[78]
	Shanxian granule	↓VEGF, MVD	Liver cancer	Liver cancer of qi-deficiency and blood-stasis	[128]

“–” means no expression

**Table 2:** Target modulators and symptomatic targets of Chinese medicine in tumour therapy

Chinese medicine		Tuning state	Target shooting					References
		State	Symptomatic target (tumour key targets and characteristics)					
		(Heat, phlegm, stagnation, deficiency) state	Tumour angiogenesis	Defective apoptosis	Tumour cell proliferation	Tumour invasion metastasis	Tumour immune escape	
Medicinal pair	Scutellaria barbata–Hedyotis diffusa	Heat	−	+	+	−	−	[129–131]
	Paridis rhizomap–Smilax glabra		−	+	+	−	−	[102, 132, 133]
	Asarum sagittarioides–Evodia rutaecarpa		−	−	+	+	+	[134–136]
	Coptis chinensis–Evodia rutaecarpa		−	+	+	+	+	[137, 138]
	Fritillaria thunbergii–Baijiezi	Phlegm	−	+	−	+	−	[138–140]
	Pinellia ternata–Aconiti Radix		−	+	−	−	+	[19, 140, 141]
	Edgaria darjeelingensis–Curcuma zedoaria	Stagnation	+	−	+	−	+	[142, 143]
	Astragali radix–Curcuma zedoaria		+	−	−	−	+	[144, 145]
	Coicis semen–Lycium chinense	Deficiency	−	−	−	−	+	[146–148]
	Bupleurum-Serpentine		−	−	−	+	−	[149]
	Psoralea corylifolia–Clinopodium megalanthum		−	+	−	−	+	[150–152]
Medicinal formula	Prescription of Qingrejiedu	Heat	+	−	−	−	+	[79]
	Yiqi Yangyin Jiedu Fang		+	−	−	−	+	[153]
	Huatan Tongyu Jiedu recipe	Phlegm	+	−	−	−	−	[124]
	Huatan Xiaoliu Decoction		+	−	−	+	−	[77]
	Xiaotan Sanjie decoction		−	−	−	+	−	[154]
	Huayu Jiedu Tongluo recipe	Stagnation	+	−	−	−	−	[126]
	Yiqi Huayu Jiedu prescription		+	−	−	−	−	[127]
	Modified Huangqi Jianzhong decoction	Deficiency	−	−	−	−	+	[78]
	Shanxian granule		−	−	−	+	+	[128]

“+” means that the Chinese medicine pair or formula plays an important role in the tumor target, “−” means that the Chinese medicine pair or formula no valid or unknown

## Summary and outlook

CM has a long history of research in tumor prevention and treatment, and clinical trials in recent decades have shown that CM is one of the most important tools for tumor treatment. Therefore, treatment by combining with the tumor inflammatory microenvironment may provide new ideas for cancer treatment.

In this paper, we summarize the tumor inflammatory microenvironment regulated by inflammatory mediators and cells, and the role of in regulating tumor inflammation. According to the theory of CM, the pathogenesis of malignant tumors lies in the invasion of external evil, dysfunction of qi and deficiency of positive qi, accompanied by states of heat, phlegm, stagnation and deficiency. Therefore, in the CM treatment of tumors, TCM is first used to regulate the imbalanced environment inside the organism, and the treatment plan is adjusted according to the actual situation of the patient to achieve the therapeutic purpose. However, the improvement of clinical physical and chemical indices of tumors by TCM treatment is still unclear.

Table 1 summarizes the role of TCM in tumor inflammation and cancer models, and lists the effects of various TCM components and formulas on cancer treatment. Table 2 summarizes the targeted regulatory effects and symptomatic targets of TCM in tumor therapy, listing the regulatory effects of representative pairs of drugs and formulas on tumor angiogenesis, apoptosis, tumor cell proliferation, tumor invasion and metastasis, and tumor immune escape. For the changes in immune cells and related factors after various TCM treatments, we can make the following conclusions: (1) Treatment with various TCM ingredients such as phytoestrogen, ginsenoside Rb1, notoginsenoside R1, astragaloside IV, platycodin D, and saponin A may have an effect on immune cells by regulating cytokines and signaling pathways. These ingredients can potentially decrease the levels of inflammatory cytokines (such as IL-6 and TNF-α), inhibit the expression of tumor-related factors (such as JAK2/STAT3, MMP-9, and vimentin), and increase immune cell activity. (2) The specific TCM ingredient combinations or formulas appear to have different effects on cellular components and factors in different states: in the Heat state, the focus is on regulating angiogenesis, promoting apoptosis defects, inhibiting tumor cell proliferation, and suppressing metastasis and immune escape; in the Phlegm state, the focus is on regulating angiogenesis, promoting apoptosis defects, and inhibiting metastasis; in the Stagnation state, the goal is to regulate angiogenesis, to promote tumor cell proliferation, and inhibit immune escape. In the Deficiency state, the focus is in inhibiting immune escape.

This article presents some research results and possible mechanisms of TCM in tumor therapy, in order to provide a reference for further research and development of TCM in cancer therapy.

## Abbreviations

TGF-β: Transforming growth factor-beta
IFN-γ: Interferon-gamma
TNF-α: Tumour necrosis factor-alpha
VEGF: Vascular endothelial growth factor
MMPs: Matrix metalloproteinases
TCM: Traditional Chinese medicine
CM: Chinese medicine
TAMs: Tumour-associated macrophages
MDSCs: Myeloid-derived suppressor cells
TANs: Tumour-associated neutrophils
DCs: Dendritic cells
IL-6: Interleukin-6
EMT: Epithelial–mesenchymal transition
IL-10: Interleukin-10
IL-17: Interleukin-17
RORγt: RAR-associated orphan receptor γt
BMDCs: Bone marrow-derived cells
IL-1β: Interleukin-1β
WT: Wild-type
SMAD: Signal transduction molecule proteins
I-Smad: Inhibitory Smad
MHC I: Primary Histocompatibility Complex Class I
SHP2: Src homologous tyrosine phosphatase 2
SOCSs: Suppression of cytokine signaling
NSCLC: Non-small cell lung cancer
Treg: Regulatory T-cells
TIMPs: Tissue inhibitors of metalloproteinases
ROS: Reactive oxygen species
ARS: Artemisinin
DHA: Dihydroartemisinin
TME: Tumour microenvironment
NETs: Neutrophil extracellular traps
